# Unc93b Induces Apoptotic Cell Death and Is Cleaved by Host and Enteroviral Proteases

**DOI:** 10.1371/journal.pone.0141383

**Published:** 2015-10-28

**Authors:** Katharine G. Harris, Carolyn B. Coyne

**Affiliations:** Department of Microbiology and Molecular Genetics, University of Pittsburgh, Pittsburgh, Pennsylvania, United States of America; Institute of Biochemistry and Biotechnology, TAIWAN

## Abstract

Unc93b is an endoplasmic reticulum (ER)-resident transmembrane protein that serves to bind and traffic toll-like receptors (TLRs) from the ER to their appropriate subcellular locations for ligand sensing. Because of its role in TLR trafficking, Unc93b is necessary for an effective innate immune response to coxsackievirus B3 (CVB), a positive-sense single stranded RNA virus belonging to the enterovirus family. Here, we show that Unc93b is cleaved by a CVB-encoded cysteine protease (3C^pro^) during viral replication. Further, we define a role for Unc93b in the induction of apoptotic cell death and show that expression of wild-type Unc93b, but not a mutant incapable of binding TLRs or exiting the ER (H412R), induces apoptosis. Furthermore, we show that cellular caspases activated during apoptosis directly cleave Unc93b. Interestingly, we show that the 3C^pro^- and caspase-mediated cleavage of Unc93b both occur within ten amino acids in the distal N-terminus of Unc93b. Mechanistically, neither caspase-mediated nor 3C^pro^-mediated cleavage of Unc93b altered its trafficking function, inhibited its role in facilitating TLR3 or TLR8 signaling, or altered its apoptosis-inducing effects. Taken together, our studies show that Unc93b is targeted by both viral- and host cell-specific proteases and identify a function of Unc93b in the induction of apoptotic cell death.

## Introduction

Unc93b is an endoplasmic reticulum (ER)-resident transmembrane protein that is required for signaling from endosomally localized Toll-like receptors (TLRs) as well as TLR5, a cell surface receptor [1–4]. TLRs are pattern recognition receptors (PRRs) that recognize pathogen associated molecular patterns (PAMPs) and initiate intracellular innate immune signaling in response to viral, bacterial, or parasitic infections [5]. Unc93b serves as a chaperone, binding its client TLRs in the ER and trafficking them to their appropriate intracellular locations where they are then available to sense their cognate ligands [6,7]. The function of Unc93b was initially discovered through a forward genetic screen in mice [1]. In this screen, mice expressing a non-functional point mutant of Unc93b, (H412R), were shown to be sensitive to a diverse group of pathogens. Unc93b was initially found to function in the trafficking of endosomally localized TLRs to the endolysosomal compartment [1,6,7], whereas Unc93bH412R is incapable of binding TLRs [6] or exiting the ER [7,8]. Recently, Unc93b was also shown to be responsible for the trafficking of a cell surface TLR, TLR5, from the ER to the plasma membrane [4]. Finally, Unc93b has been implicated in TLR-independent inflammasome activation in response to bacterial RNA [9]. From these studies, a more complete understanding of the function of Unc93b is emerging that suggests that Unc93b is a central regulator of a number of key innate immune pathways involved in pathogen detection and clearance.

The importance of Unc93b and its client TLRs in initiating immune responses to viral infections was underscored when it was discovered that children inheriting two autosomal recessive mutant alleles of Unc93b that produce a non-functional, truncated version of the protein developed Herpes Simplex Encephalitis (HSE), a rare but serious viral encephalopathy, after Herpes Simplex Virus-1 (HSV-1) infection [10]. An increased risk of HSE after HSV-1 infection is also seen in children with autosomal dominant mutations in TLR3 [11], suggesting that the trafficking of TLR3 by Unc93b is crucial for the control of HSV-1 infection. Additionally, it was shown that patients with systemic lupus erythematosus (SLE) had higher levels of Unc93b expressed in immune cells than in healthy control patients, suggesting that high levels of Unc93b are responsible for the dysregulated TLR signaling known to be associated with SLE pathogenesis [12]. Thus, Unc93b is important in both innate immune defense against pathogens and in the development of autoimmunity in humans.

In addition to TLR-mediated signaling, the induction of cell death is a powerful innate immune pathway by which host cells protect themselves from microbial infections. Indeed, many components of TLR-mediated signaling also participate in the induction of pro-apoptotic signaling in response to viral infections, underscoring the importance of these pathways in host defenses. For example, the TLR3-associated adaptor molecule TIR-domain-containing adapter-inducing interferon-β (TRIF) potently induces apoptosis via its receptor homotypic interacting motif (RHIM) domain located in its C-terminus [13,14].

Coxsackievirus B3 (CVB) is a member of the enterovirus family; these are small (~30nm), non-enveloped, positive-sense single stranded RNA viruses among the most common human viral pathogens worldwide [15,16]. CVB is a leading cause of myocarditis, with up to 35% of myocarditis cases being associated with CVB infections [17]. TLR3 is likely the critical TLR involved in detecting and mounting cellular responses to CVB infections *in vivo*. Humans who develop myocarditis after enteroviral infections have increased frequencies of single nucleotide polymorphisms (SNPs) in TLR3 that render them less responsive to TLR3 ligands [18], and TLR3^-/-^ mice are more susceptible to CVB infection and exhibit increased mortality and myocarditis [19]. Taken together, these data suggest that TLR3 senses the dsRNA intermediate produced during CVB replication, and that this sensing and subsequent activation of innate immune signaling is key to the control of CVB infection. Indeed, recent work has shown that *Unc93b1* ^*LETR/LETR*^ mice, which have a loss of function mutation in the gene encoding Unc93b, exhibit increased mortality upon CVB-induced myocarditis due to higher viral titers and dysregulation of inflammation in these mice [20].

CVB utilizes cell death-mediated destruction of the host cell membrane for its egress/spread. However, the virus must control cell death induction precisely during its replication as activating cell death prematurely, or by alternative pathways, could inhibit replication and/or induce inflammatory signaling. The induction of caspase-mediated apoptosis is the most common means by which CVB facilitate its egress [21–23]. However, the mechanisms by which CVB modulates cell death signaling are likely complex and involve the alteration of many components associated with cell death signaling pathways. CVB commonly alters host cell signaling, including TLR and cell death signaling, by the direct cleavage of host proteins by the 2A^pro^ and 3C^pro^ virally encoded cysteine proteases [24,25], which often renders these proteins nonfunctional or alters their functions in ways meant to enhance viral replication.

Interestingly, analysis of microarray data [20] using DAVID [26,27] from cardiac tissue of wild-type mice versus *Unc93b1* ^*LETR/LETR*^ mice found that there was a significant enrichment in the expression of genes involved in cell death pathways in CVB-infected wild-type animals (p = 0.0051), suggesting that Unc93b might play a role in cell death signaling during CVB infection. In addition, given that Unc93b has been directly linked to CVB-induced pathogenesis *in vivo*, we examined whether Unc93b was involved in cell death signaling during infection. Here, we show that ectopic expression of Unc93b robustly induces apoptosis, and that this induction requires its exit from the ER as the H412R mutant of Unc93b lacking this function is incapable of inducing cell death. In addition, we show that caspases activated by TNFα signaling, or by the induction of TRIF-mediated TLR3 signaling, directly cleave Unc93b at a single site (position D27) located within its N-terminus. Additionally, we show that the CVB-encoded protease 3C^pro^ also cleaves Unc93b at a single site located within 10 amino acids of the caspase cleavage site (at position Q17). Collectively, our findings point to a previously uncharacterized role for Unc93b in the initiation of apoptosis during TLR3 signaling and suggest that both host and viral proteases target the N-terminus of Unc93b to alter some aspect of its function.

## Materials and Methods

### Cells and Virus

Human kidney 293T cells and human osteosarcoma U2OS cells were purchased from the American Type Culture Collection (ATCC). Human kidney 293 cells stably expressing Bcl-XL and HA-tagged TLR8 were purchased from Invivogen. Human kidney 293T cells stably expressing Flag-tagged TLR3 were kindly provided by Dr. Saumendra Sarkar (University of Pittsburgh). All cells were cultured in DMEM supplemented to contain 10% FBS and 1x penicillin/streptomycin. Experiments were performed with CVB3-RD (expanded as previously described in [28]). CVB3-RD was UV-inactivated for 30 minutes using a Spectrolinker XL-1000.

### Plasmids

WT Unc93b constructs were constructed by amplification of Unc93B1 pCR4-TOPO purchased from Harvard PlasmID (clone ID: HsCD00341813) with primers encoding a C-terminal Myc tag followed by insertion into the HindIII and KpnI sites of eGFP-C2 (NT-GFP, CT-Myc construct) or pcDNA3.1(+) (CT-Myc construct). Truncated Unc93b constructs were constructed by amplification of Unc93B1 pCR4-TOPO using primers encoding a C-terminal Myc tag and beginning amplification at residues 18 or 28 for Δ17 or Δ27, respectively, followed by insertion into the HindIII and KpnI sites of pcDNA3.1(+). Unc93bΔ30 was constructed by amplification of Unc93B1 pCR4-TOPO using primers encoding a C-terminal Myc tag and beginning amplification at residue 31 followed by insertion into the HindIII and KpnI sites of eGFP-C2. Unc93b N-terminal fragments were constructed by amplification of Unc93B1 pCR4-TOPO using primers encoding a stop codon after residue 17 or 27 for NT17 or NT27, respectively, followed by insertion into the HindIII and KpnI sites of eGFP-C2. Point mutations producing alanine substitutions in residues Q17 and D27 or an arginine substitution in residue H412 were generated by splice overlap extension, as previously described [29], primer sequences available upon request. NT-GFP, CT-Myc Unc93bQ17A or Unc93bD27A were then constructed by amplification using primers encoding a C-terminal Myc tag followed by insertion in the HindIII and KpnI sites of eGFP-C2. CT-Myc Unc93bH412R was then constructed by amplification using primers encoding a C-terminal Myc tag followed by insertion into the HindIII and KpnI sites of pcDNA3.1(+).

Myc-tagged 3C^pro^ constructs (WT and C147A) were generated as previously described [24,30]. TRIF constructs were generated as previously described [24]; NT-TRIF contains residues 1–359, whereas CT-TRIF contains residues 360–712. Venus VSV-G was purchased from Addgene (plasmid 11914, deposited by Dr. Jennifer Lippincott-Schwartz and described previously [31]).

### Reagents

Poly(I:C) was purchased from Invivogen (tlrl-pic) and used at 1μg/mL. R848 was purchased from Invivogen and used at 10 μg/mL zVAD-fmk was purchased from Calbiochem (Caspase Inhibitor VI), Invivogen (tlrl-vad), or ApexBio (A1902) and was used at 100 μM. TNFα was purchased from Sigma (H8916) and was used at 50 ng/mL. JSH-23 was purchased from Calbiochem (481408) and was used at 7.1 μM. Staurosporine was purchased from Sigma (S6942) and was used at 8 μM.

### Antibodies

Mouse monoclonal and rabbit polyclonal antibodies against GFP (B-2, sc-9996), GAPDH (FL-335, sc-25778 or sc-25778 HRP), and Myc (sc-789) were purchased from Santa Cruz Biotechnology. Rabbit monoclonal antibody against PARP (46D11, CST-9532) was purchased from Cell Signaling Technology. Mouse monoclonal anti-enterovirus VP1 antibody (Ncl-entero) was obtained from Novocastra Laboratories. Goat antibodies against rabbit or mouse IgG and conjugated to HRP were purchased from Santa Cruz Biotechnology.

### Transfections

DNA plasmid transfections were performed using Xtreme Gene HP DNA transfection reagent (Roche) according to manufacturer’s protocol with modification for reverse transfection. In some cases, forward transfections were performed according to manufacturer’s protocol.

### Western Blots

Cells were lysed in Luciferase Cell Culture Lysis Reagent (CCLR; Promega; 25 mM Tris-phosphate pH 7.8, 2mM DTT, 2mM 1,2-diaminocyclohexane-N,N,N’,N’-tetraacetic acid, 10% glycerol, 1% Triton X-100) or in some cases, RIPA buffer. Lysates were sonicated and insoluble material was cleared by centrifugation. Lysates were boiled at 95°C for approximately 10 minutes with SDS-Sample Buffer (Boston BioProducts), loaded into 4–20% Criterion Tris-HCl precast polyacrylamide gels, separated electrophoretically, and transferred to nitrocellulose membranes. Membranes were probed with indicated primary antibodies diluted 1:1000 in 5% milk in PBS-T at room temperature (RT) for 1 hour or at 4°C overnight. Following washing in PBS-T, membranes were incubated with appropriate horseradish peroxidase-conjugated secondary antibodies (Santa Cruz Biotechnology) diluted 1:10000 in PBS-T for 30 minutes at RT. Membranes were washed in PBS-T and signal was detected using SuperSignal West Pico, Dura, or Femto chemiluminescent substrates (Pierce Biotechnology).

### Reporter Gene Assays

Cells were cotransfected with a plasmid containing a firefly luciferase gene under the control of an IFNβ promoter (p125-luc) and a plasmid containing a renilla luciferase gene (pRL-null) at a ratio of 30:1. Luciferase was quantified using the Dual Luciferase Reporter assay system (Promega) and a Synergy 2 luminescence plate reader (Bio-Tek).

### Immunofluorescence Microscopy

Confluent monolayers grown in 8-well chamber slides (BD Biosciences) were fixed in 4% paraformaldehyde and permeabilized with 0.1% Triton X-100. Cells were incubated with the indicated primary antibodies for 1 hour at RT, washed in PBS, then incubated in appropriate AlexaFluor-conjugated secondary antibodies for 30 minutes at RT, washed in PBS, and mounted in Vectashield containing DAPI (Vector Labs). Images were captured using a FV1000 confocal laser scanning microscope (Olympus). Image analysis was performed using Imaris and the module ImarisColoc. For differential interference contrast (DIC) microscopy, confluent monolayers were grown in 35 mm glass-bottomed dishes (Mat Tek) and images were captured from living cells using IX81 or IX83 inverted microscopes (Olympus).

### Cell Titer Glo Assays

Cells to be analyzed by Cell Titer Glo Assay were grown to confluency in 96-well plates, then mock- or staurosporine-treated. Cell Titer Glo Luminescent Cell Viability Assays (Promega) were performed as described in manufacturer’s protocol.

### Flow Cytometry

Cells were grown to confluence in 24-well plates, then stained with Annexin V and Propidium Iodide (PI) using the AlexaFluor488 Annexin V/Dead Cell Apoptosis Kit (Invitrogen) according to the manufacturer’s protocol and analyzed using a MACSQuant Analyzer (Miltenyi). Data analysis was performed using FlowJo software.

### Statistical Analysis

Student’s t-tests or one-way or two-way ANOVAs were performed as appropriate using GraphPad Prism. * = p≤0.05, ** = p≤0.01, *** = p≤0.001, **** = p≤0.0001. Data are shown as mean ± standard deviation.

## Results

### Unc93b expression induces cell death

**Fig 1:**
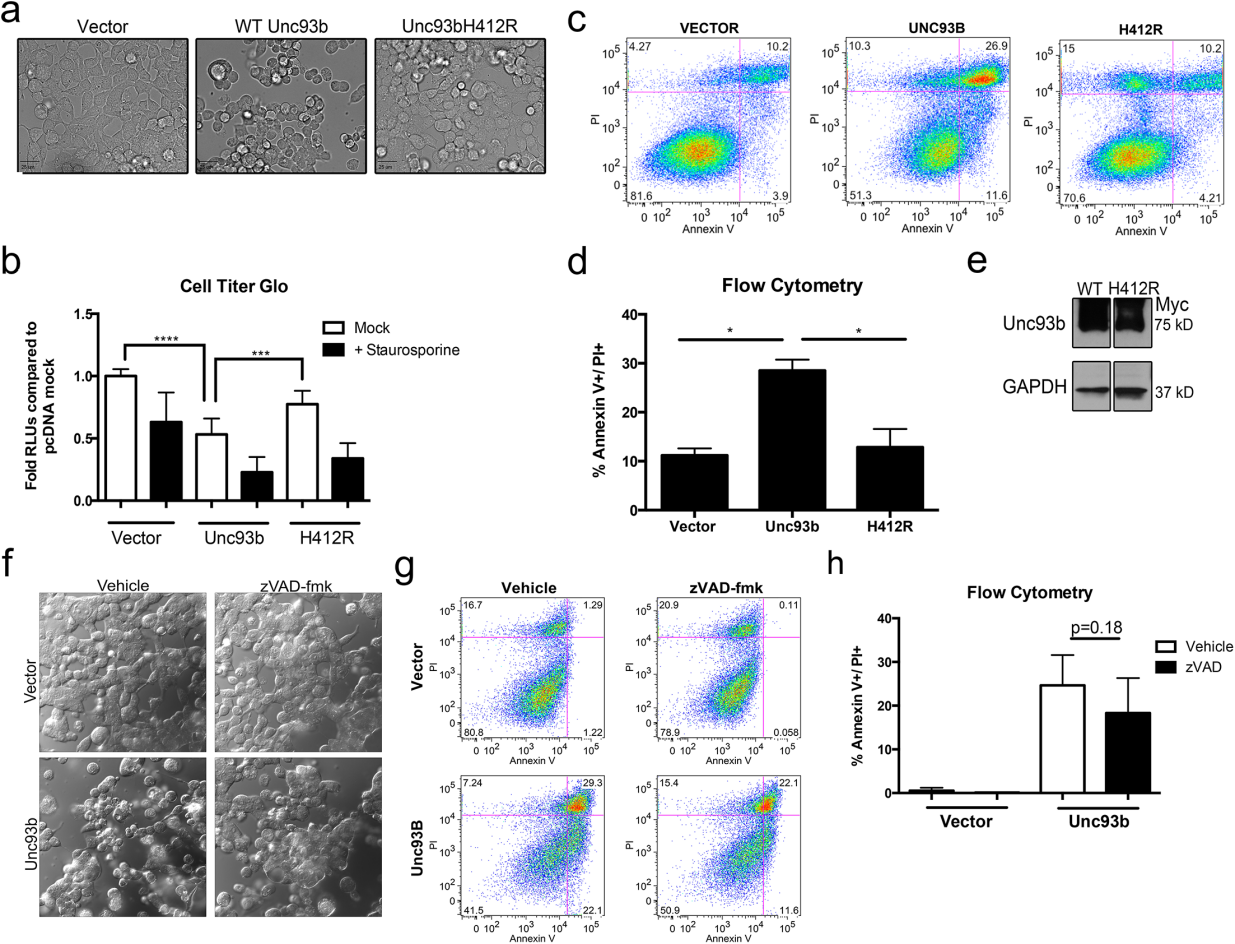
Unc93b expression induces apoptotic cell death. (**a-h**) 293T cells were transfected with WT or H412R Unc93b containing a C-terminal Myc tag (**a**) 48 hours post-transfection, differential interference contrast (DIC) images were obtained from live cells. (**b**) 48 hours post-transfection, cells were treated with staurosporine for 24 hours and cell death was measured by Cell Titer Glo assay. Data shown is an average of 6 experiments. (**c**) 72 hours post-transfection, cells were stained with Annexin V and PI and analyzed by flow cytometry. Data shown are representative of 4 experiments. Cell population was determined by gating events on Forward Scatter (FSC) and Side Scatter (SSC). Cells were then gated based on Annexin V and PI staining intensities. (**d**) 48–72 hours post-transfection, cells were stained with Annexin V and PI and analyzed by flow cytometry as in 1c, and Annexin V+/PI+ populations were compared. Data shown are representative of 4 experiments (**e**) 293T cells were transfected in parallel with those shown in 1d, lysates were harvested and then subjected to immunoblotting with anti-Myc or -GAPDH antibodies. (**f**) Prior to transfection (1 hour) cells were treated with zVAD-fmk and then transfected with the indicated constructs for 48 hours (in zVAD-fmk-containing medium). Approximately 48 hours post-transfection, DIC images were obtained from live cells. (**g,h**) Prior to transfection (1 hour) cells were treated with zVAD-fmk and then transfected with the indicated constructs for 72 hours (in zVAD-fmk-containing medium). 72 hours post-transfection cells were stained with Annexin V and PI and analyzed by flow cytometry as in 1c. Data shown are representative of 2 experiments (**g**) or average of 2 experiments (**h**).

The overexpression of several components associated with innate immune signaling, including the TLR3 adaptor TRIF, potently induces apoptosis [13,32,33]. We found that ectopic expression of Unc93b in 293T cells consistently induced significant morphological changes in transfected cells, resulting in cell rounding, disruption of cell monolayers, and other signs of cell stress/death (Fig 1A). To further investigate these morphological changes, we measured cell viability based on quantitation of ATP through a luminescent detection method (Cell Titer Glo) and found that Unc93b overexpression significantly decreased cell viability (Fig 1B). Additionally, we found that Unc93b expression also enhanced cell death induced upon treatment with staurosporine (Fig 1B). To identify the cell death pathway(s) initiated by Unc93b, we determined the impact of Unc93b overexpression on propidium iodide (PI) uptake and Annexin V binding by flow cytometry. We found that Unc93b specifically induced apoptotic cell death and led to an ~3-fold increase in the levels of PI- and Annexin V-positive cells. (Fig 1C and 1D). Additionally, we found that the induction of cell death was partially sensitive to caspase inhibition, as treatment of cells with the pan-caspase inhibitor zVAD-fmk was sufficient to block Unc93b induced morphological changes associated with cell death and caused a mild reduction in PI- and Annexin V-positive cells that did not reach statistical significance (Fig 1F, 1G and 1H). Of note, the frequency of PI-positive cells was not reduced by zVAD-fmk treatment, suggesting that upon blockage of caspase-dependent pathways, alternative pathways may be activated (data not shown).

The H412R mutant of Unc93b has been well characterized for its inability to bind TLRs [6], facilitate TLR signaling [1], or traffic out of the ER [7,8]. To determine whether these functions were also required for the apoptosis-inducing properties of Unc93b, we determined the effect of Unc93b H412R expression on apoptosis using the assays described above. Importantly, we found that the H412R mutant of Unc93b did not induce morphological changes when overexpressed in 293T cells (Fig 1A), did not alter cellular ATP levels (Fig 1B), and did not induce apoptosis as assessed by flow cytometry, despite equivalent levels of expression (Fig 1C, 1D and 1E). Taken together, these data implicate a previously uncharacterized role for Unc93b in the induction of apoptosis and suggest that its ability to traffic from the ER is required for this function.

### Unc93B is cleaved by caspases

**Fig 2:**
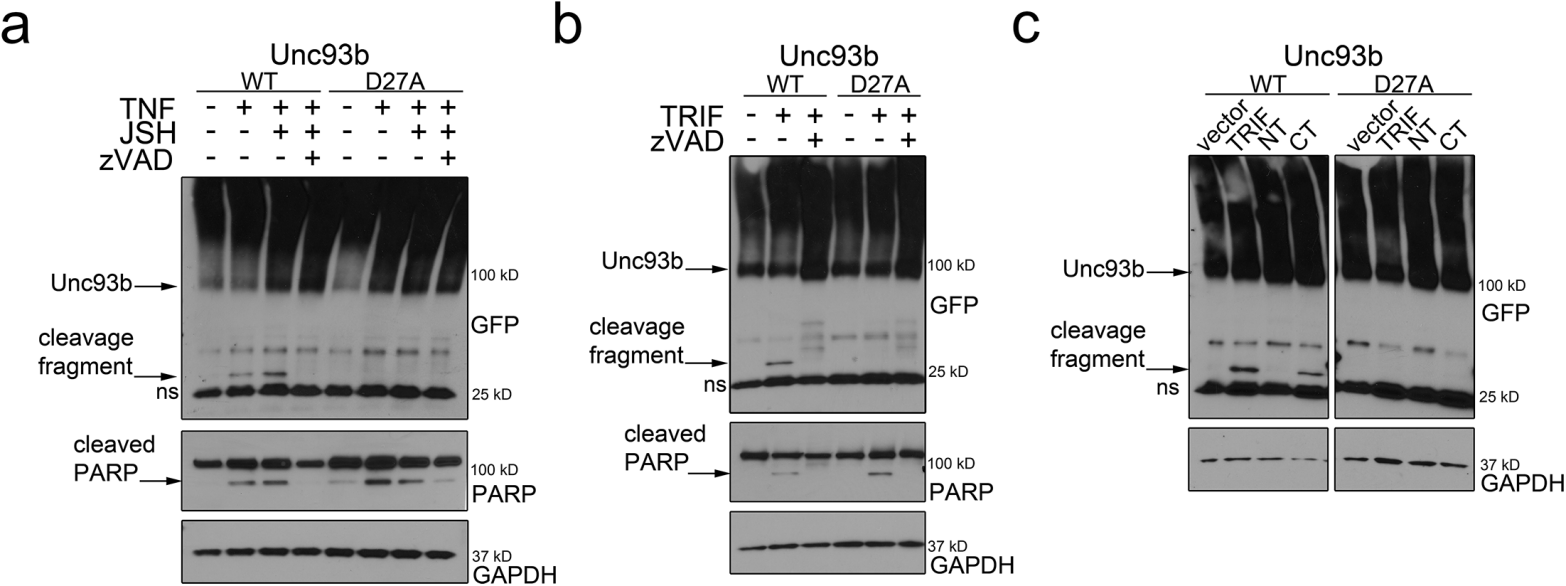
Unc93b is cleaved by caspases during apoptosis. (**a**) 293T cells were transfected with the indicated EGFP-fused Unc93b construct and 48 hours post-transfection, cells were treated with TNFα, JSH-23, and Z-VAD-FMK (or mock control) for 18 hours. Lysates were harvested and subjected to immunoblotting with anti-GFP, -PARP, or -GAPDH antibodies. (**b**) 293T cells were transfected with the indicated EGFP-fused Unc93b constructs and Ha- Flag dual-tagged TRIF and treated with zVAD-fmk 6 hours post-transfection. Lysates were harvested 48 hours post-transfection and subjected to immunoblotting with anti-GFP, -PARP, and–GAPDH antibodies. (**c)** 293T cells were transfected with the indicated EGFP-fused Unc93b and Flag-tagged TRIF constructs (Full-length, N-terminal or C-terminal); lysates were harvested 48 hours post-transfection and subjected to immunoblotting with anti-GFP and -GAPDH antibodies. In all panels, ns denotes nonspecific bands.

Because we observed a role for Unc93b in the induction of apoptosis, we next examined whether Unc93b is regulated by caspase-mediated cleavage during apoptotic cell death as a negative feedback mechanism to attenuate this function. To do this, we activated caspases via TNFα treatment and determined whether this treatment led to the proteolytic cleavage of Unc93b. For these studies, we utilized an N-terminal EGFP fusion construct of Unc93b overexpressed in 293T cells. We found that activation of caspases through TNFα treatment induced the appearance of an ~33 kD N-terminal GFP-fused cleavage fragment of Unc93b when assessed by immunoblotting (Fig 2A). The quantity of this cleavage fragment was enhanced when cells were treated with TNFα in the presence of the nuclear factor kappa-light-chain-enhancer of B cells (NF-κB) inhibitor JSH-23 (Fig 2A). By immunoblotting, Unc93b appears as a major species band and a high molecular weight ‘smear’, consistent with reports that Unc93b is ubiquitinated [34]. Importantly, Unc93b cleavage was inhibited by the addition of a pan-caspase inhibitor, zVAD-fmk, confirming the role of caspases in this cleavage event (Fig 2A). Caspase activation was confirmed by detection of cleaved poly ADP ribose polymerase (PARP), a classical caspase substrate [35] (Fig 2A). Using a caspase cleavage site detection algorithm [36,37], we identified two potential caspase cleavage sites (D21 and D27) within the N-terminus of Unc93b that would induce the appearance of the ~33kD N-terminal GFP-fused cleavage product detected by immunoblotting. We found that site-directed mutagenesis of D27 to alanine (D27A) completely inhibited the caspase-dependent cleavage of Unc93b (Fig 2A), thus demonstrating that D27 is the site in Unc93b targeted by caspases.

Sustained signaling through TLR3 can result in caspase-mediated cell death [38]. As Unc93b is necessary for TLR3 signaling, we postulated that TLR3-dependent caspase activation might also result in Unc93b cleavage via the activation of caspases. To test this, we overexpressed the TLR3 adaptor protein TRIF, which induces TLR3 signaling, and found that TRIF overexpression resulted in Unc93b cleavage in a caspase dependent manner at residue D27 (Fig 2B). TRIF-mediated caspase activation has been shown to be dependent on its C-terminal RHIM domain [13]. To determine whether this domain was required for the TRIF-induced cleavage of Unc93b, we expressed an N-terminal construct of TRIF lacking the RHIM domain (NT) or a C-terminal construct containing the RHIM domain (CT). We found that expression of the C-terminus of TRIF alone was sufficient to induce Unc93b cleavage and that this cleavage occurred at position D27 as cleavage was absent in the D27A mutant of Unc93b (Fig 2C). Collectively, these data show that Unc93b is targeted by cellular caspases at position D27 during apoptosis induced by TNFα or by TLR3-TRIF signaling.

### Unc93b is cleaved by the CVB virally-encoded protease 3C^pro^

**Fig 3:**
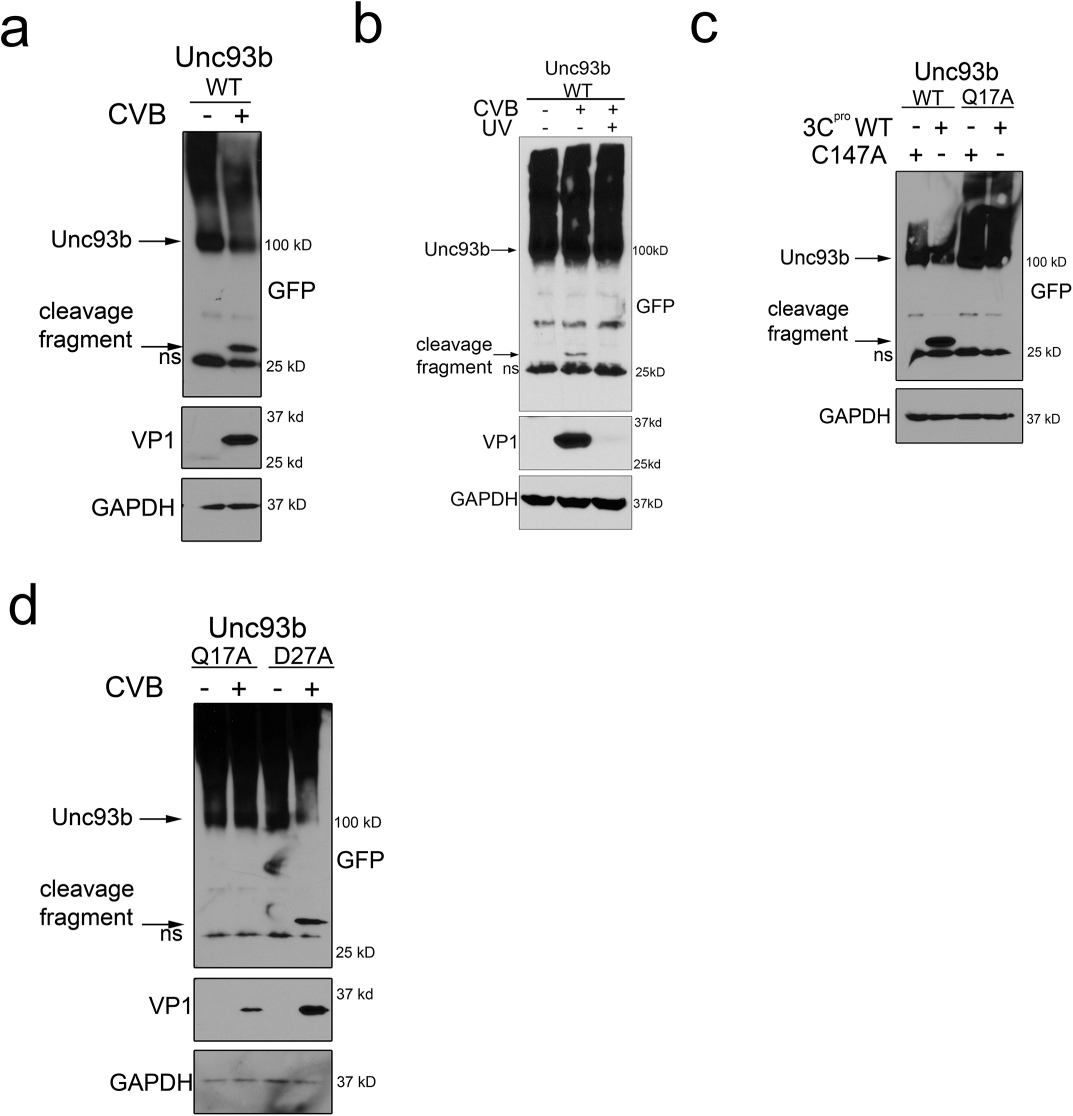
Unc93b is cleaved by 3C^pro^ during CVB infection. **(a)** 293T cells transfected with EGFP-Unc93b were infected with CVB (MOI = 1), lysates harvested 18 hours post-infection, then subjected to immunoblotting with anti-GFP, -VP1, and -GAPDH antibodies. (**b**) 293T cells transfected with EGFP-Unc93b were infected with CVB or UV-inactivated CVB (MOI = 1), lysates harvested 18 hours post-infection, then subjected to immunoblotting with anti-GFP, -VP1, and -GAPDH antibodies. **(c)** 293T cells were cotransfected with the indicated EGFP-fused Unc93b construct and Myc-tagged wild-type (WT) or catalytically inactive C147A 3C^pro^, lysates were harvested 48 hours post-transfection and subjected to immunoblotting with anti-GFP and -GAPDH antibodies. (**d**) 293T cells transfected with the indicated EGFP-fused Unc93b construct were infected with CVB (MOI = 1), lysates were harvested 16 hours post-infection and subjected to immunoblotting with anti-GFP, -VP1, and–GAPDH antibodies. In all panels, ns denotes nonspecific bands.

Because we found that caspases cleaved Unc93b during apoptotic cell death signaling, we next assessed whether this cleavage might also occur during CVB infection given that this virus often utilizes the direct cleavage of cellular innate immune and/or cell death signaling components by virally-encoded proteases to facilitate its replication. Using 293T cells overexpressing N-terminal EGFP-fused Unc93b, we detected the appearance of an ~30 kD N-terminal GFP-fused cleavage fragment of Unc93b in CVB-infected cells by immunoblotting (Fig 3A). The appearance of this fragment required active viral replication, as we did not observe Unc93b cleavage when cells were infected with UV-inactivated virus (Fig 3B). Although mutagenesis of residue D27 protected Unc93b from caspase-mediated cleavage, we found that the D27A mutant of Unc93b was not protected from CVB-induced cleavage, suggesting that other viral and/or cellular factors were responsible for cleavage of another site(s) within Unc93b (Fig 3D). CVB is known to utilize virally encoded proteases to target host proteins [39]. Using an algorithm designed to detect the semi-conserved consensus cleavage sites for the two CVB-encoded proteases 2A^pro^ and 3C^pro^ [40], we identified a putative 3C^pro^ cleavage site in Unc93b at residue Q17, which would be predicted to produce an N-terminal GFP-fused fragment consistent with that observed during CVB infection. In contrast, Unc93b did not contain any consensus 2A^pro^ cleavage sites. We found that overexpression of wild-type (WT) CVB 3C^pro^, but not a catalytically inactive mutant lacking protease activity (C147A), induced the appearance of the ~30kD N-terminal cleavage fragment of EGFP-fused Unc93b (Fig 3C). By performing site-directed mutagenesis, we found that 3C^pro^ specifically targeted residue Q17 in the N-terminus of Unc93b as a mutant of this site (Q17A) completely resisted cleavage (Fig 3C). We confirmed that Q17 was the sole site targeted during CVB infection by infecting 293T cells expressing either the Q17A or caspase-resistant D27A Unc93b mutants and assessing their cleavage sensitivity during infection. (Fig 3D). Taken together, these data suggest that Unc93b is targeted for cleavage by the virally-encoded 3C^pro^ CVB protease during CVB infection.

### Unc93b cleavage does not affect TLR signaling or Unc93b trafficking

**Fig 4:**
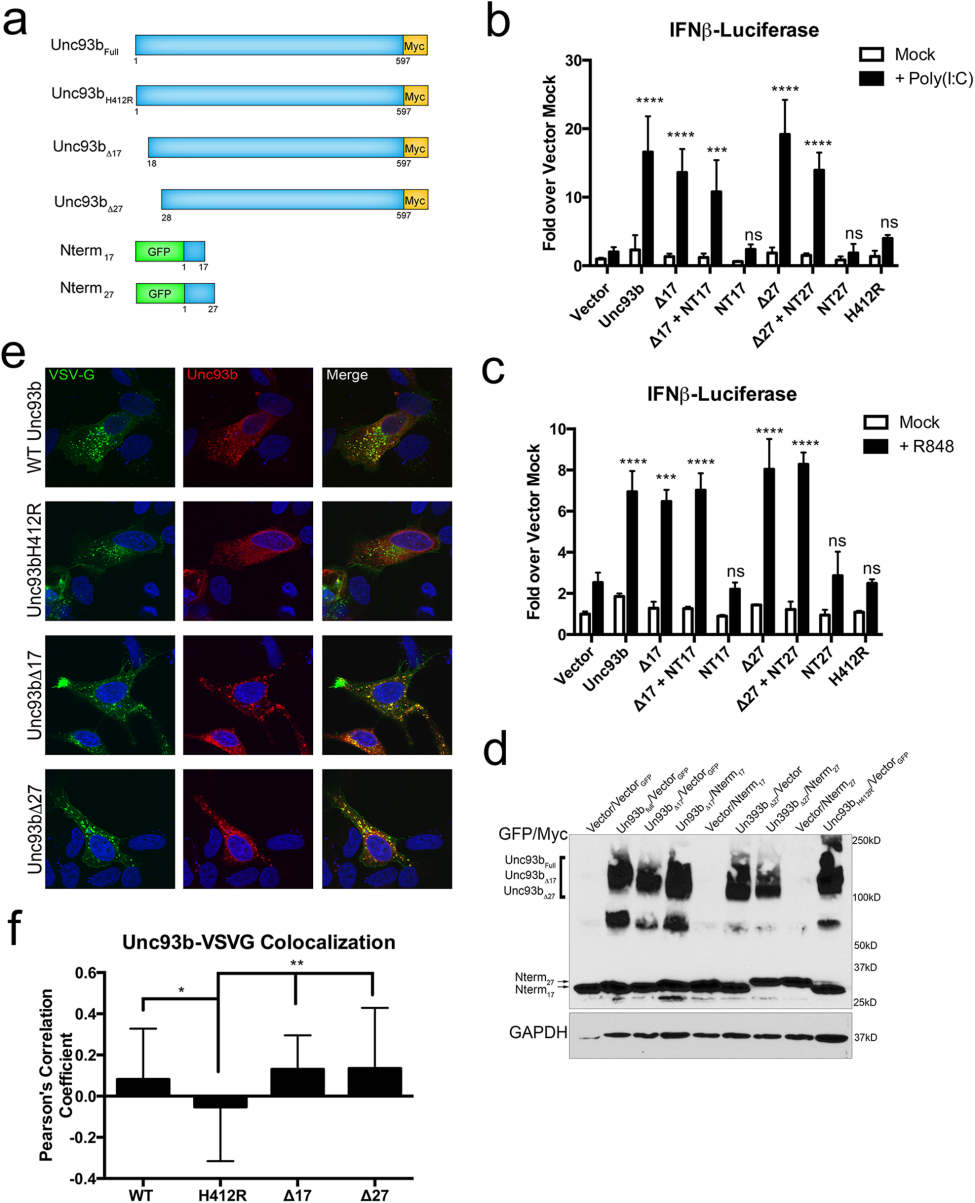
Unc93b cleavage does not affect TLR signaling or secretory pathway trafficking. (**a**) Schematic of wild-type Unc93b (Unc93b_Full_), Unc93b-H412R mutant (Unc93b_H412R_), Unc93b truncation mutants (Unc93bΔ17 or Un93bΔ27) and Unc93b cleavage fragments (Nterm_17_ and Nterm_27_). (**b, c**) 293T-FlagTLR3 (b) or HEK293XL-TLR8HA (c) cells were co-transfected with the indicated Unc93b construct containing a C-terminal Myc tag and/or N-terminal GFP tag, and IFNβ luciferase reporter (pIFNβ-fluc) and control renilla (pRL-null) constructs. Approximately 24 hours post transfection, cells were treated with 1 μg/mL poly(I:C) (b) or 10 μg/mL R848 (c) for 16 hours. Luciferase levels were analyzed using the Dual Luciferase reporter assay system. Firefly luciferase levels were normalized to renilla luciferase levels and then normalized to the vector transfected mock-treated condition. Data shown are an average of 2 independent experiments (b) or a representative experiment (c) and significance is compared to vector plus ligand. (**d**) Immunoblotting of lysates collected from 293T cells used in panel 4c and probed with antibodies against GFP and Myc (top) or GAPDH (bottom) as a loading control. (**e, f**) U2OS cells were co-transfected with Venus-fused VSV-G and the indicated Unc93b constructs containing a C-terminal Myc tag. Approximately 48–72 hours post-transfection, cells were fixed and then immunostained for Myc and confocal microscopy performed (**e)** and then images analyzed to obtain Pearson’s correlation coefficients (**f**).

As TLR3 and TLR8 signaling have been established as an important step in the initiating an immune response to CVB, we next assessed the possibility that 3C^pro^-mediated and/or caspase-mediated cleavage of Unc93b affects its ability to facilitate TLR3/8 signaling. To do this, we generated truncation mutants of Unc93b missing the first 17 or 27 N-terminal amino acids (Unc93bΔ17 and Unc93bΔ27, schematic Fig 4A), which is representative of the C-terminal fragment of Unc93b that remains in the cell following cleavage by 3C^pro^. Additionally, we generated fragments of Unc93b consisting of only the first 17 or 27 N-terminal amino acids fused to an N-terminal GFP tag (Unc93bNT17 and Unc93bNT27) (Fig 4A). We then determined the effect of expression of these truncation mutants on IFNβ induction downstream of TLR3 and TLR8, endosomal TLRs entirely dependent on Unc93b for their endosomal trafficking [6,7]**.** We first treated 293T or HEK293 cells stably expressing TLR3 or TLR8, and transiently expressing a firefly luciferase construct downstream of the IFNβ promoter, with poly(I:C) or R848, synthetic TLR3 or TLR8 ligands, respectively. We then examined activation of the TLR signaling pathways in the presence or absence of Unc93b and Unc93b truncation mutants by measuring luciferase levels. We found that expression of WT Unc93b enhanced both TLR3 and TLR8 signaling, whereas Unc93bH412R did not (Fig 4B and 4C **)**. Additionally, both Unc93bΔ17 and Unc93bΔ27 enhanced TLR3 and TLR8 signaling, similar to WT Unc93b (Fig 4B and 4C), indicating that the ability of Unc93b to facilitate TLR signaling is not affected by 3C^pro^- or caspase-mediated cleavage. We also found that the co-expression of the N-terminal fragments of Unc93b, Nterm_17_ and Nterm_27_, that would be generated by 3C^pro^- or caspase-mediated cleavage, respectively, with Unc93bΔ17 or Unc93bΔ27 did not alter TLR signaling in any way (Fig 4B and 4C), confirming that cleavage of Unc93b is unlikely to negatively impact TLR signaling. Efficiency of transfection of the indicated Unc93b mutants/fragments was monitored by immunoblotting (Fig 4D).

We identified two distinct mechanisms of Unc93b cleavage—one dependent on a virally-encoded protease during viral infection and one induced by cellular caspases. Both of these cleavage events sever the N-terminal tail of Unc93b before its first transmembrane domain. Given that two unrelated proteases cleave Unc93b, we next examined the effect of 3C^pro^- and caspase-mediated Unc93b cleavage on a hallmark Unc93b function: its ability to traffic through the secretory pathway. We overexpressed WT Unc93b, Unc93bΔ17, Unc93bΔ27, or Unc93bH412R in U2OS cells with EGFP-fused VSV-G, a glycoprotein from vesicular stomatitis virus widely used in the study of intracellular trafficking [31]. As expected, WT Unc93b efficiently trafficked through the secretory system, as indicated by its co-localization with VSV-G in punctate structures (Fig 4E and 4F). However, Unc93bH412R failed to localize to these VSV-G positive punctae (Fig 4E and 4F), supporting previously published data indicating that Unc93bH412R is incapable of exiting the ER [7,8]. Similar to WT Unc93b, both Unc93bΔ17 and Unc93bΔ27 co-localized efficiently with the VSV-G positive punctae (Fig 4E and 4F), indicating that the trafficking of Unc93b is unaffected by 3C^pro^- and caspase-mediated N-terminal truncations.

### Unc93b cleavage does not affect its ability to induce cell death

**Fig 5:**
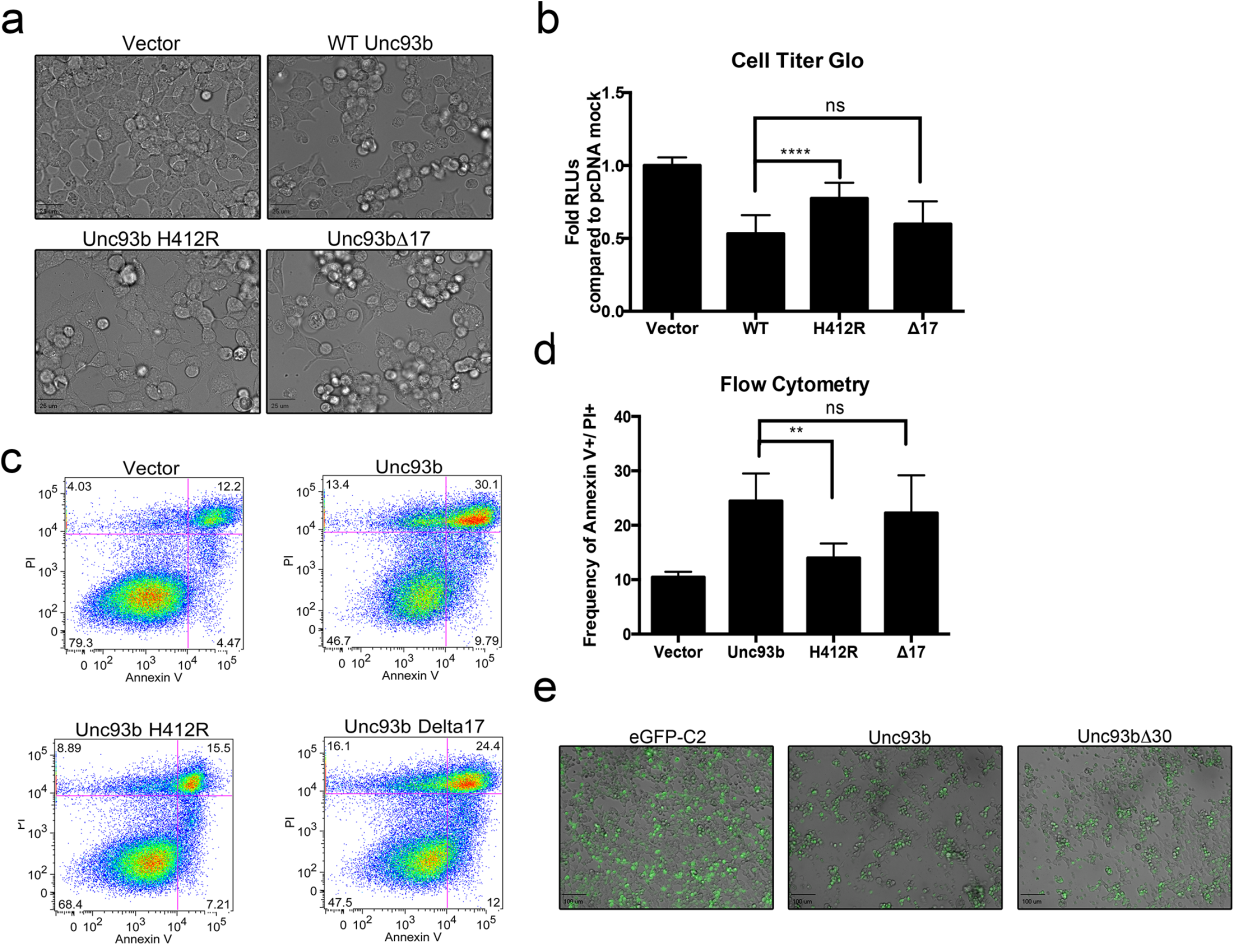
Unc93b cleavage does not affect its induction of cell death. **(a-d)** 293T cells were transfected with the indicated Unc93b construct containing a C-terminal Myc tag. (**a**) 72 hours post-transfection, differential interference contrast (DIC) images were obtained from live cells. (**b**) 48 hours post-transfection, cell death was measured by Cell Titer Glo assay. Data shown is average of 6 experiments. (**c**) 72 hours post-transfection, cells were stained with Annexin V and PI and analyzed by flow cytometry. Data was analyzed as described in Fig 1C, gating events on FSC vs. SSC for cell population are not shown here. Data shown are representative of three experiments. (**d**) 48–72 hours post-transfection, cells were stained with Annexin V and PI and analyzed by flow cytometry. Data were analyzed as described in Fig 1C, and Annexin V+/PI+ populations were compared. Data shown are average of three separate experiments (**e**) 293T cells were transfected with eGFP-fused Unc93b constructs containing a C-terminal Myc tag. 48–72 hours post-transfection, DIC images were obtained from live cells.

Because we found that ectopic expression of Unc93b induced apoptosis, we next assessed whether the 3C^pro^- and/or caspase-induced cleavage of Unc93b would ablate this function. We found that Unc93bΔ17 promoted apoptotic cell death at a level comparable to that of WT Unc93b as observed morphologically (Fig 5A) and measured by Cell Titer Glo and flow cytometric analysis as described previously (Fig 5B–5D). In addition, an N-terminal truncation mutant lacking the first thirty amino acids (Δ30) of Unc93b also induced morphological changes consistent with apoptosis, suggesting that the N-terminal cleavage of Unc93b by caspases also does not affect this function (Fig 5E). Although the cells used in this study (293T) express low levels of endogenous Unc93b, it is unlikely that these endogenous levels are sufficient to mask any lack of functionality in the truncation mutants given that ectopic expression of Unc93bH412R did not induce apoptotic cell death.

## Discussion

Unc93b is critical for an effective immune response to diverse pathogens, including CVB, and has been associated with the development of autoimmune disease. Despite its important role in human health, its function has only recently been uncovered and, accordingly, our understanding of the complete functions of Unc93b has been rapidly expanding in both breadth and depth. Here we show that CVB utilizes its virally-encoded 3C^pro^ cysteine protease to directly target the distal N-terminus of Unc93b. Similarly, we show that cellular caspases also target this region and cleave Unc93b within 10 amino acids from the site of 3C^pro^ cleavage. In addition, we further broaden the known role of Unc93b by describing its function in initiating apoptotic cell death pathways and find that the H412R mutant of Unc93b is unable to induce cell death.

Here, we describe a new function for Unc93b in the induction of apoptotic cell death. As it has been previously shown that genes involved in cell death are downregulated in the cardiac tissue of mice expressing a non-functional Unc93b variant [20], it is likely that Unc93b is involved in the maintenance of a balance between cell death and cell survival under resting conditions. Additionally, cell death induction is critical in the response to many intracellular pathogens [41]. Thus, further understanding the role Unc93b is playing in cell death helps to broaden the understanding of Unc93b function during initiation of innate immune and cell death signaling. Although it remains unclear by what mechanism Unc93b initiates apoptosis, it is intriguing that Unc93bH412R is incapable of inducing this pathway. Unc93bH412R is incapable of binding TLRs, but the cell type used in our study (293T) does not express TLRs at significant levels [42]. Thus, the role that Unc93b plays in inducing cell death is most likely independent of its role in TLR trafficking and/or signaling. In addition to its inability to bind TLRs, Unc93bH412R also remains localized to the ER [8] and does not localize to endolysosomal vesicles. Given the lack of apoptosis induction by Unc93bH412R and its retention in the ER, it is possible that Unc93b may be involved in a more general trafficking pathway than previously appreciated, and serves to traffic components of apoptotic signaling out of the ER. Although the precise mechanism(s) by which Unc93b induces apoptosis remain to be determined, our work indicates that the effect is at least partially mediated by cellular caspases as the morphological changes associated with Unc93b-mediated apoptotic cell death are inhibited by the pan-caspase inhibitor zVAD-fmk.

The caspase-mediated cleavage of Unc93b discovered here serves to place Unc93b into the broader context of interactions between innate immune proteins and caspases. In recent years, caspases have been shown to be closely involved in the regulation of innate immune signaling through execution of specific cleavage events. For instance, innate immune signaling upon activation of retinoic acid-inducible gene (RIG)-I, a cytosolic RNA sensor, is dependent on recruitment of the serine threonine nonreceptor kinase receptor-interacting protein (RIP)-1. Caspase-8 serves to cleave and disable RIP1, providing a negative feedback mechanism to control and down-regulate innate immune signaling [43]. Conversely, caspase activation has also been reported to be required for activation of innate immune signaling, as NF-κB activation can be dependent on Caspase-8 activity [44–46]. Clearly, the role of caspase-mediated cleavage events is crucial not only in the context of programmed cell death, but also in non-apoptotic functions associated with the regulation of intracellular innate immunity. Thus, it seems likely that the caspase-mediated cleavage of Unc93b serves to alter its role in some aspect of host innate immune and/or cell death signaling.

It is of special note that two evolutionarily independent proteases, the virally encoded 3C^pro^ cysteine protease of CVB and host caspases, target Unc93b for cleavage within the same 10 amino acid N-terminal region. As caspase activation results in the cleavage of Unc93b at residue D27, we initially suspected that this cleavage event would serve to modulate the proapoptotic signaling role of Unc93b. However, we found that Unc93bΔ30 was fully capable of inducing cell death under the conditions we tested. Although we found that truncated versions of Unc93b produced by both 3C^pro^ and caspases were capable of performing the known functions of Unc93b, we suspect that this important and under-characterized protein has more functions than previously elucidated, and it is likely that these cleavage events serve to hinder one of those functions. We eagerly anticipate the advancement of the field of research surrounding Unc93b and expect that soon these cleavage events will be placed in their proper functional context.
